# Structural Dynamics of Human Telomeric G-Quadruplex Loops Studied by Molecular Dynamics Simulations

**DOI:** 10.1371/journal.pone.0071380

**Published:** 2013-08-08

**Authors:** Hong Zhu, Shiyan Xiao, Haojun Liang

**Affiliations:** 1 CAS Key Laboratory of Soft Matter Chemistry, Department of Polymer Science and Engineering, University of Science and Technology of China, Hefei, Anhui, People's Republic of China; 2 Hefei National Laboratory for Physical Sciences at Microscale, University of Science and Technology of China, Hefei, Anhui, People's Republic of China; Bioinformatics Institute, Singapore

## Abstract

Loops which are linkers connecting G-strands and supporting the G-tetrad core in G-quadruplex are important for biological roles of G-quadruplexes. TTA loop is a common sequence which mainly resides in human telomeric DNA (hTel) G-quadruplex. A series of molecular dynamics (MD) simulations were carried out to investigate the structural dynamics of TTA loops. We found that (1) the TA base pair formed in TTA loops are very stable, the occupied of all hydrogen bonds are more than 0.95. (2) The TA base pair makes the adjacent G-quartet more stable than others. (3) For the edgewise loop and the diagonal loop, most loop bases are stacking with others, only few bases have considerable freedom. (4) The stabilities of these stacking structures are distinct. Part of the loops, especially TA base pairs, and bases stacking with the G-quartet, maintain certain stable conformations in the simulation, but other parts, like TT and TA stacking structures, are not stable enough. For the first time, spontaneous conformational switches of TTA edgewise loops were observed in our long time MD simulations. (5) For double chain reversal loop, it is really hard to maintain a stable conformation in the long time simulation under present force fields (parm99 and parmbsc0), as it has multiple conformations with similar free energies.

## Introduction

G-quartet is a layer formed by four guanines held together with eight hydrogen bonds. In guanine-rich nucleic acid sequences, the formation of several consecutive G-quartets can form inter-molecular or intra-molecular four-stranded structures, termed G-quadruplexes. Telomeres, at the end of most eukaryotic chromosomes, usually comprise simple tandem repeats of guanine rich sequences which can form non-canonical DNA conformations like quadruplexes. Maintenance of the quadruplex structure in telomere has been regarded as a target for anticancer drug discovery, and has been investigated widely [1], [2].

Loops which are linkers connecting G-strands and supporting the G-tetrad core exist in all of the intra-molecular G-quadruplexes and most of the inter-molecular G-quadruplexes. Loops can be divided into three typical families: (1) edgewise loops connecting two adjacent guanines; (2) diagonal loops connecting two opposite guanines in the G-quartet; and (3) double chain reversal loops run across the G-quadruplex grooves, from one side to another side of G-quadruplex stems.

Loops are important for biological roles of G-DNA molecules. For instance, thrombin binding aptamer (TBA) is a 15-mer DNA oligonucleotide which can fold into a G-quadruplex with two TT loops and a TGT loop. Recent researches demonstrated that TT loops participate in direct binding to thrombin, and the function of the TGT loop is to stabilize the aptamer structure [3], [4]. The sequence of loops has influence to the stability of G-quadruplexes. If TTA loops of hTel are replaced by AAA, the G-quadruplex stability decreases largely [5]. Similar results also found in TBA's TT loops, when thymine residues are substituted by adenine, the G-quadruplex is more stable or more unstable than that of wild-type base on the position of the thymine which is been replaced [6]. Besides, the loop length and loop sequence affect the folding topologies and stability of G-quadruplexes, which have been investigated by experimental and computational methods [7], [8]. In some cases, the behavior of loops also affects the process of ligands binding to the G-quadruplex. Hexaoxazole-containing macrocycle derivative ligands bind to hTel G-quadruplex inducing an abnormal increase of the system entropy [9], . The possible reason is that the loop base stacking structure is destroyed by the ligands binding process which inducing the increase of conformational entropy.

MD simulations have been applied in investigating G-quadruplex for a long time [11], [12]. Some significant works have done in recent years. For instance, the formation and stability of G-triplex DNA [13], [14], and folding pathways of hybrid type hTel [15]. In another work, the relative stabilities of varied glycosidic conformations of G-tracts were compared with free energy analysis [16]. This work is particularly important as it unveiled some folding rules of the G-quadruplex.

Structural dynamics of the loops, which connect to its biological roles, also investigated by MD simulations. MD simulations combined with simulated annealing (SA) and locally enhanced sampling methods (LES) have been used to predict the favorable topologies and conformations of TT (T2) and TTT (T3) loops [17]. It concluded that even though the favored type of loops accords with the experimental results, but the predicted loop conformations are distinct with experimental structures. The predicted optimal structure of TTTT (T4) loop also differs from the experiments [18]. The failure is due to the misbalance of the AMBER parm99 force field [19] which has been used in these works. The AMBER parmbsc0 force field [20], which refined the 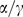 torsion from parm99 force field, has been confirmed to be valid for DNA duplex in hundreds of nanoseconds state-of-the-art molecular dynamics simulations in aqueous solution [21]. A recent research also demonstrated that the parmbsc0 provides G-quadruplexes loops conformations that are clearly closest to the experiments [22].

To our knowledge, another type of loop sequence, which contains adenine base, like TTA, has not been widely researched yet. One distinction between this type and loops which contain only thymine base (like T3 and T4 loops mentioned above) is that the former one has a chance to form TA Watson-Crick base pair, and this base pair may stabilizes the loop effectively. So its behavior may be very different with T3 loops. Recently, A microsecond time scale MD simulation on propeller hTel G-quadruplex shows that the conformational space of loops is rich and more dynamic than we have assumed to date [23]. This work stressed that simulation time is important in sampling conformational space of G-quadruplexes. Actually, hTel sequence can fold into different types of topologies, including anti-parallel basket structure [24], parallel structure [25], [3+1] hybrid structure [26], [27] and some higher-order structures [28]. In the present work, we chose two other topologies of hTel, anti-parallel structure and hybrid structure, and focused on three topics: (1) the stability of TA base pair in TTA loops; (2) the dynamical behavior of different TTA loops and (3) the contribution of TA base pair to the stability of the G-stem.

## Results

**Figure 1:**
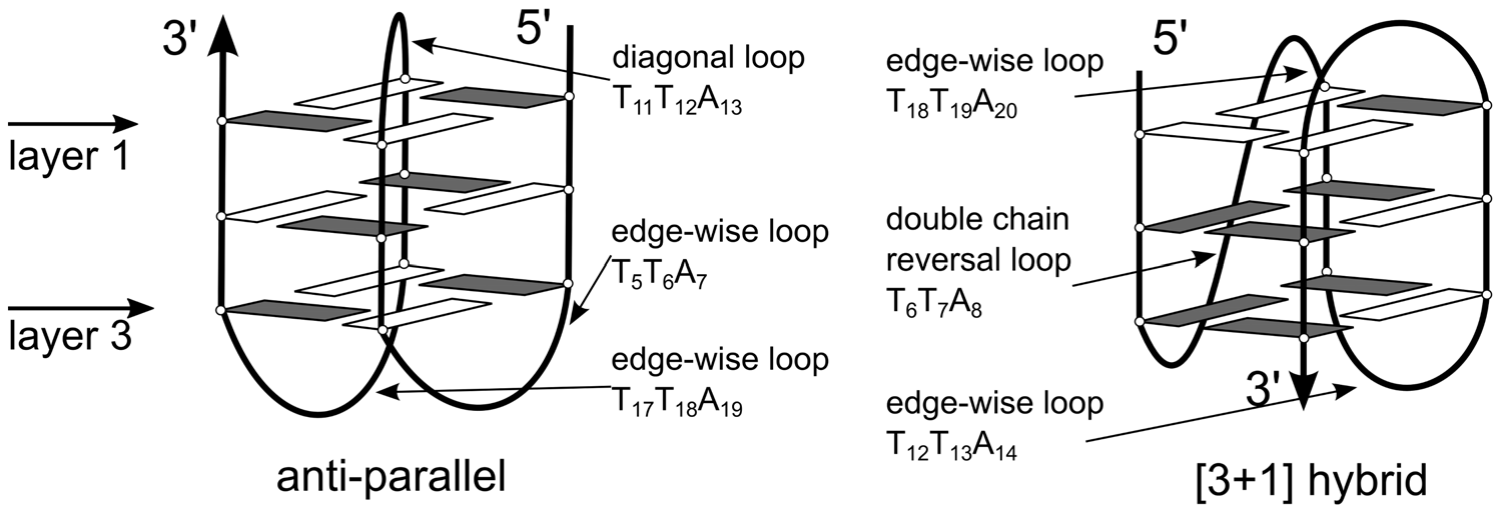
Scheme of two hTel G-quadruplex structures involved in this work. *Syn* and *anti* glycosidic bond orientations are drawn in white and gray. From layer 1 to layer 3 are three G-quartets named from the orient of the first strand of G-quadruplexes.

In the present work, we chose anti-parallel structure (PDB code, 143D) and [3+1] hybrid structure (PDB code, 2GKU) for simulations (Figure 1). The first one has the sequence d[AG
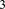
(T
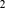
AG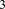)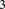], and another one has the sequence d[T
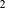
G
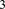
(T
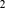
AG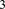)
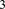
A]. These two structures contain all the three typical types of loop conformations. Simulations and abbreviations are listed in Table 1.

**Table 1:** List of simulations involved in this work.

PDB ID	ions type	force field	time(ns)	abbreviation
143D	Na^+^	parm99	1000	anti_99
2GKU	K^+^	parm99	1000	hybrid_99
143D	Na^+^	parmbsc0	1000	anti_bsc0
2GKU	K^+^	parmbsc0	1000	hybrid_bsc0
143D	Na^+^	parmbsc0	5	anti_stem^a^
2GKU	K^+^	parmbsc0	5	hybrid_stem^a^

a In these two structures, loop bases are deleted and just G-quartets left.

**Figure 2:**
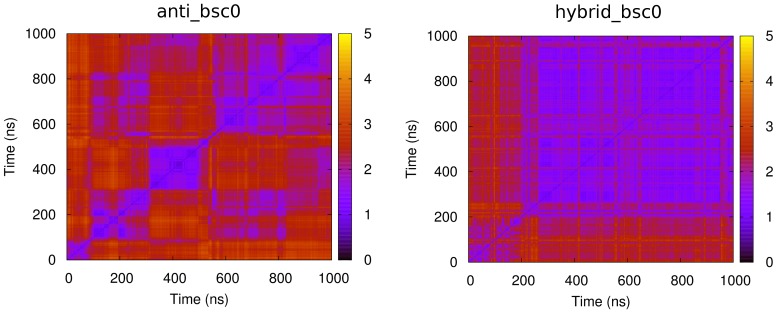
Structural dynamics of anti-parallel and hybrid type hTel G-quadruplex on different time scales assessed by pairwise RMSD matrices.

In order to identify the importance of time scale on the structural dynamics of these two G-quadruplexes, the pairwise root mean square deviation (RMSD) was assessed (Figure 2). One principal result is that in both structures, the conformational fluctuation has the time scale of hundreds nanoseconds. Besides, the two structures have different forms of fluctuations. In the anti-parallel structure, the initial conformation maintained in the first 100 ns, and then two other conformations each maintained 200 ns, another conformation maintained in the last 400 ns. In contrast, the hybrid structure settled down to a conformation after 200 ns. These primary results indicate that in order to characterize the structural dynamics of G-quadruplex loops, the simulation time should be as long as several hundred nanoseconds. It coincides with a recent work [23]. In addition, the RMSD of all G-stems were in the range of 1 Å with tiny fluctuations (Figure 8), which means that the G-stem of all models were stable and maintained their initial structure in the simulations. It accords with other works on similar G-quadruplexes in dozens of nanoseconds under the same force field [22], [29]. So the conformation change mainly happened in loops, and more detailed discussion about loops was given in the next subsections.

### Behavior of Loops in Anti-parallel Structure

#### Description of the Structure

There are two edgewise loops (T
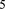
T
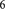
A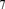 and T
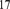
T
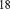
A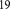) and a diagonal loop (T
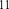
T
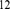
A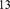) in the anti-parallel hTel G-quadruplex. In the crystal structure, A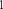 and A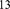 planes are approximately parallel to the adjacent G-quartet plane with A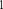 stacking over the adjacent G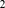 and A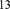 stacking over the cross strand G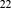. A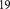 is parallel to the adjacent quartet and cross-strand stacks over G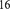, while T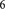 is parallel to and stacks over non-adjacent G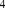. The A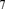 base exhibits a pronounced tilt relative to the adjacent quartet plane and is the only adenine that does not stack with a guanine in this G-quadruplex [24].

#### Behavior of the Upper Diagonal Loop T
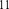
T
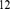
A
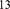


**Figure 3:**
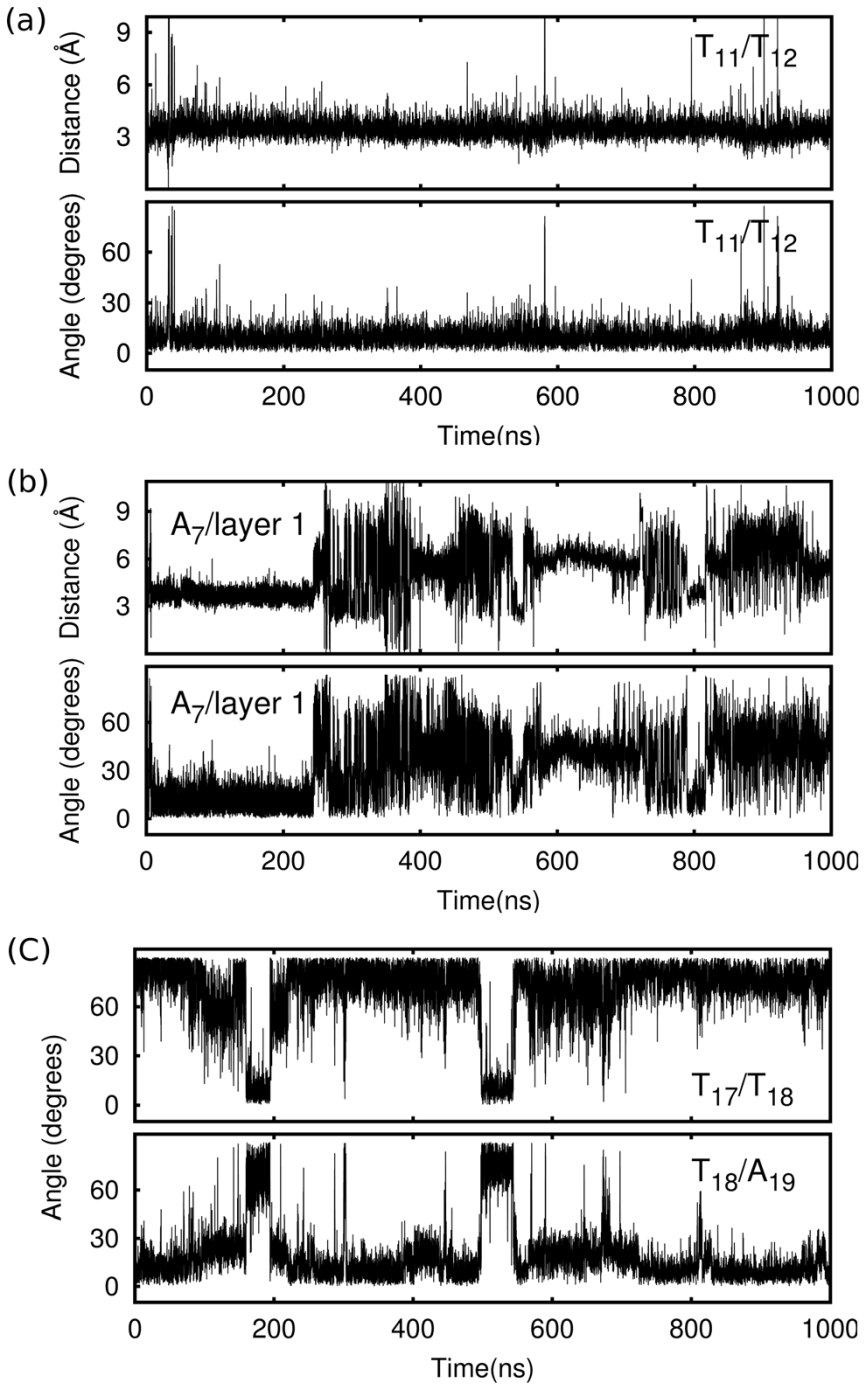
Structural dynamics of anti_bsc0 loops. (a), distance and angle between two thymine bases T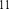 and T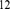 of the upper diagonal loop. The small fluctuation reflects that these two thymine bases stacked with each other in the trajectory. (b), distance and angle between A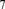 and upper G-quartet, in the first 250 ns, A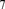 stacked with the G-quartet, but then this stacking structure was destroyed. (c), angle between T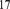 and T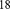 and angle between T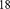 and A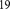.

**Figure 4:**
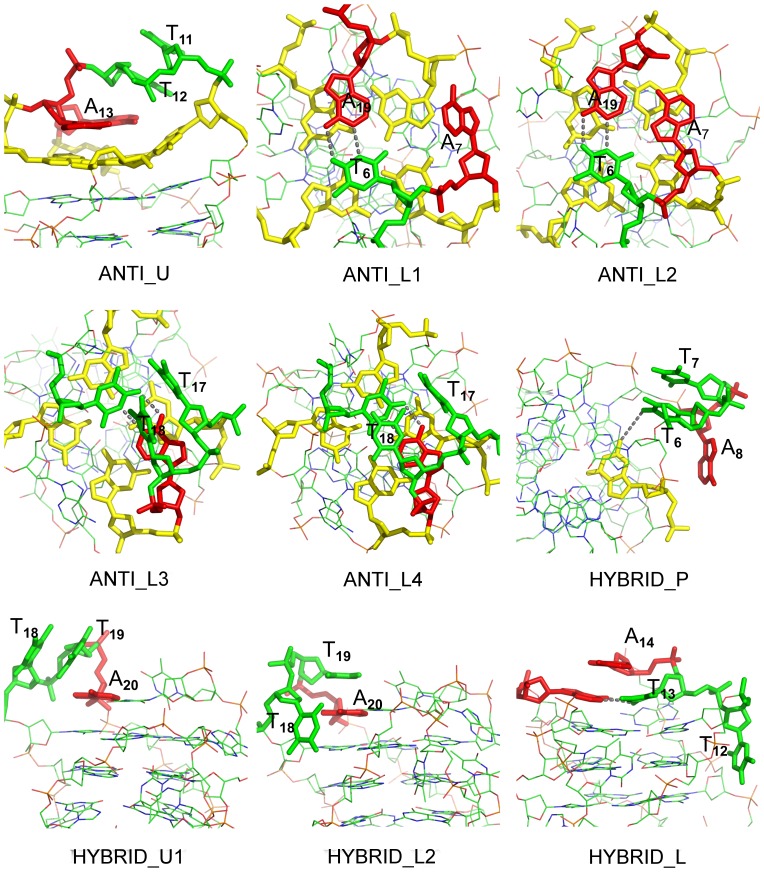
TTA loops conformations discussed in this work. Thymine in blue, adenine in green and guanine in yellow. Hydrogen bonds are drawn in gray dash lines, and all bonds connected with hydrogen are hidden.

There is a diagonal loop T
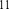
T
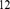
A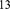 and a single terminal base A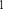 on the upper side of anti-parallel structure. In the simulation, two adenine bases A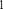 and A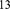 were stacking with adjacent G-quartet with considerable stability (distance and angle between adenine base and adjacent G-quartet are listed in Table 2). By monitoring the distance and angle between two thymine bases T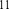 and T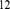, we found that they stacked with each other in the trajectory (Figure 3a), and also found that this stacking conformation only maintained in anti_bsc0, but did not stable enough to maintain in anti_99 (Figure 9a). Furthermore, these two residues also had a considerable fluctuation as a whole, and this fluctuation led to the deviation of the adjacent quartet bigger than other two quartets (this loop conformation is named ANTI_U, Figure 4).

**Table 2:** Distance and angle between loop bases and their reference base groups^a^.

Model	Group 1	Group 2	Distance(Å)	Angle(degree)
anti_bsc0	A_1_	layer 1	3.2±0.3	21.9±9.3
	A_13_	layer 1	2.8±0.7	22.9±11.4
	T_6_	layer 3	2.8±0.5	14.8±7.2
	A_19_	layer 3	3.1±0.4	14.6±6.3
hybrid_bsc0	T_1_	layer 1	2.6±0.7	13.7±5.8
	A_20_	layer 1	3.0±0.3	9.0±5.0
	T_13_	layer 3	2.5±0.5	20.3±9.0
	A_24_	layer 3	2.7±0.4	16.1±7.5
	A_14_	T_13_/A_24_	4.8±0.7	27.4±10.7

Results under parm99 force field are given in Table S1.

a The adjacent G-quartet or adjacent base pair are chosen as the reference base group.

#### Behavior of the Lower Edgewise Loops T
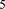
T
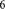
A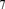 and T
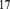
T
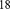
A
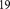


There are two edgewise loops T
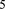
T
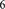
A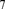 and T
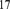
T
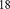
A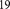 on the lower side of anti-parallel structure. Among them, A canonical Watson-Crick DNA base pair formed with two hydrogen bonds between T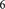 and A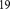. This base pair was very stable, and the occupied of both hydrogen bonds were more than 0.95 (Table 3). In addition to this, T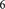 and A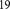 were stacking with the lower layer of G-quartet. The small fluctuation of distance and angle between T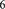/A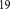 and adjacent G-quartet manifests that this stacking structure is very stable (Table 2).

**Table 3:** Details of hydrogen bonds between loop bases and between loop base and quartet base.

Model	Donors	Acceptors	Occupied(%)	lifetime(ps)
anti_bsc0	N6@A19	O4@T6	98.3	322.5
	N3@T6	N1@A19	98.4	441.5
	N2@G8	O4′@A_7_	91.4^a^	101.0
hybrid_bsc0	N3@T13	N1@A24	99.2	1229.5
	N6@A24	O2@T13	99.0	659.8
	N3@T1	N1@A20	99.9	6989.5
	N6@A20	O4@T1	99.4	931.0
	N2@G4	O4@T6	79.2	96.0

Results under parm99 force field are given in Table S2.

a This hydrogen bond formed after 250 ns in the simulation, so just last 700 ns of the trajectory was used to count the hydrogen bond.

In the NMR structure, A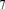 is the only adenine does not stack with adjacent G-quartet. However, it was stacking with the adjacent G-quartet after the energy minimization step of the simulations (the conformation is named ANTI_L2, Figure 4). This stacking conformation maintained hundreds of nanoseconds (100 ns in anti_99 and 250 ns in anti_bsc0, Figure 3b and Figure 9b), and then it was destroyed and formed a conformation (named ANTI_L1, Figure 4) similar to the NMR structure. The angle between A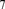 and adjacent G-quartet was 40±17 degrees in anti_bsc0 and 42±18 degrees in anti_99. A hydrogen bond formed between O4′ of A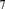 and N2 of G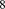 to help stabilizing this conformation (occupied of the hydrogen bond is about 0.91, Table 3). It reflects that the loop conformation is the competition of diverse force, like stacking interaction and hydrogen bond. MM/PBSA results indicated that ANTI_L1 is the more stable conformation with free energy 18 kcal/mol lower in anti_basc0 (Table 4).

**Table 4:** Comparison of the absolute free energies^a^ of the different conformations of the loops.

Model	Conformations	ΔE*_int_*	ΔE*_eelc_*	ΔE*_vdw_*	ΔE*_sp_*	ΔE*_nsp_*	ΔPB	ΔTS	ΔG
anti_bsc0	ANTI_L1-ANTI_L2	−9	85	−4	−91	0	−19	−1	−18
anti_bsc0	ANTI_L4-ANTI_L3	−7	−14	2	17	0	−2	4	−6
hybrid_bsc0	HYBRID_L1-HYBRID_L2	13	−90	−12	57	1	−32	4	−36

Results under parm99 force field are given in Table S3.

a ΔE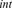, internal energy, ΔE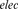, Coulombic energy, ΔE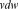, van der Waals energy, ΔE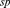, polar solvation energy, ΔE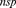, nonpolar solvation energy, ΔPB, enthalpy, ΔTS, solute entropy, ΔG, absolute free energy. All are in kcal/mol.

In T
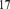
T
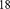
A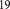 loop, A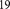 was stacking with the adjacent G-quartet, T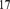 was vertical to the adjacent G-quartet and stabilized by a hydrogen bond between O4 of T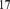 and N2 of G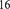, and T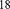 had two conformations, one is stacking with T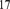 (named ANTI_L3, Figure 4) and another is stacking with A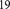 (named ANTI_L4, Figure 4). In most of the time, T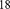 was stacking with A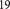, and T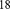 stacking with T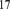 was found mainly from 100 ns to 200 ns and from 500 ns to 600 ns (Figure 3c). MM/PBSA analysis manifested that two conformations have similar free energies, the free energy difference is just 6 kcal/mol in anti_bsc0 (Table 4). Furthermore, it indicates that ANTI_L4 is more stable than ANTI_L3, which means that T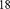 inclines to stack with A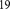. It accords with the result of our observation (T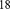/A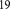 stacking conformation was found in most of the snapshots, 91% in anti_bsc0 and 80% in anti_99.). Analyzing dihedral angles of the backbone indicated that the conformational switch of T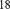 connects to its 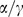 torsion. The 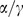 switched from *g+/t* to *g−/g+* when T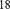 turn to stacking with T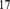 in anti_99, but changed from *t/g+* to *g−/g+* in anti_bsc0 as *g+/t* is penalized in parmbsc0.

Among these loop bases, only T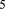 had the biggest fluctuation in the simulation, even though T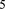 formed some weak hydrogen bonds with neighbor backbone atoms.

### Behavior of Loops in [3+1] Hybrid Structure

#### Description of the Structure

The [3+1] hybrid hTel G-quadruplex contains one double chain reversal loop (T
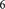
T
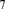
A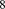) and two edgewise loops (T
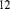
T
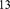
A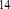 on the top and T
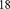
T
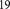
A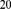 on the bottom of the G-tetrad core). The first thymine and the adenine of the double chain reversal loop have the similar positions to the crystal parallel structure, but the second thymine has the different conformation. In addition, there are Watson-Crick base pair T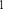/A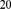 and reversed Watson-Crick base pair T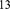/A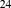 stacks on two sides of the G-tetrad [26].

#### Behavior of the Double Chain Reversal Loop T
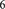
T
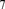
A
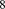


**Figure 5:**
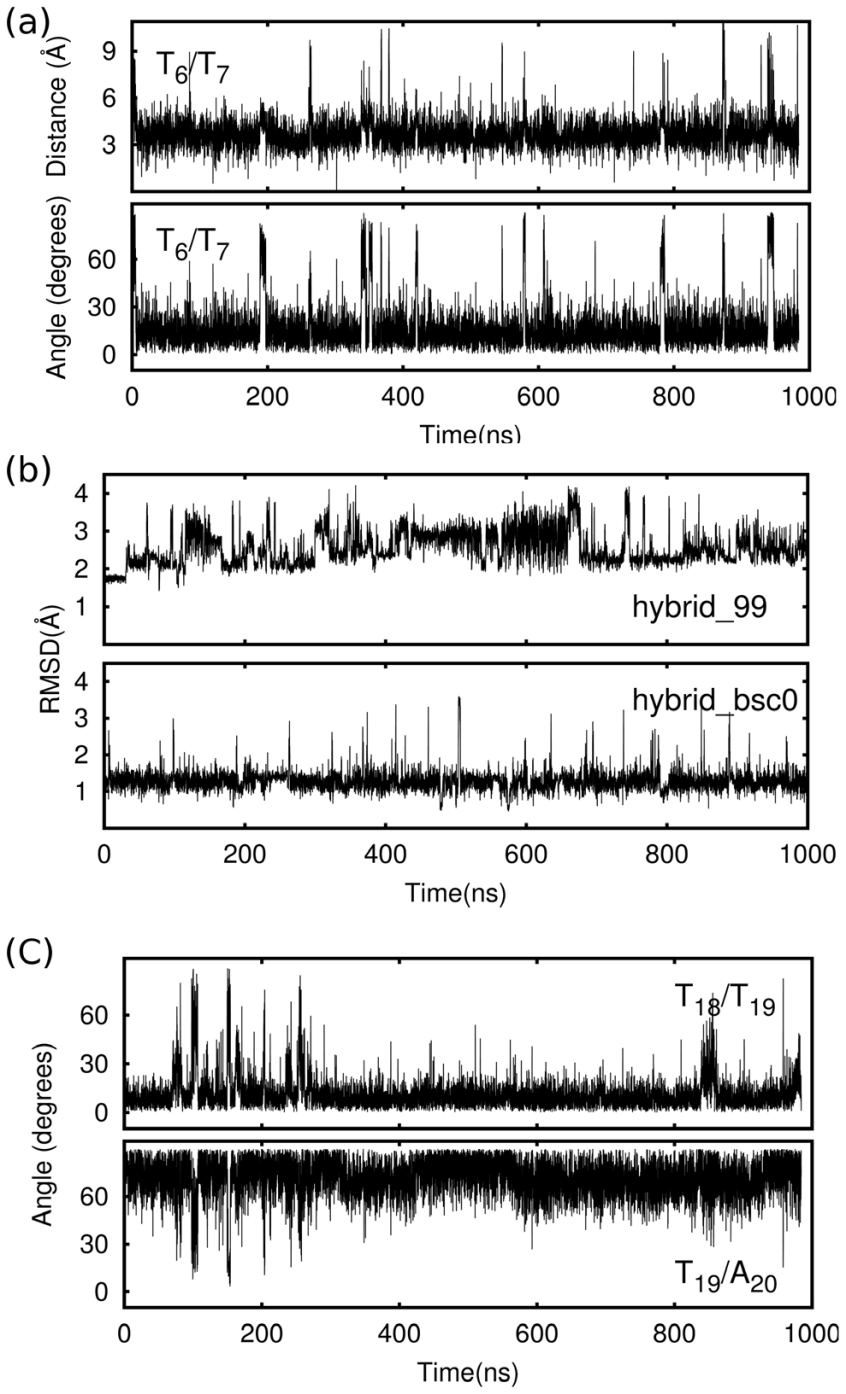
Structural dynamics of hybrid_bsc0 loops. (a), distance and angle between two thymine bases T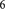 and T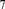 of double chain reversal loop. (b), The RMSD of T
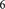
T
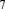
A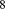 loop in [3+1] hybrid structure, and the NMR experimental structure was chosen as reference. (c), angle between T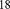 and T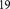 and angle between T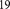 and A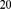.

The double chain reversal loop was flexible and did not maintain a single stable structure in the simulation. In detail, there was a hydrogen bond formed between O4 of the first thymine and middle quartet of G-stem (G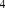). It is the same with which found in previous simulations for hTel parallel G-quadruplex structure [22]. This hydrogen bond maintained in hybrid_bsc0 in most of the time as its occupied is 79%, but it just existed in 43% of the time in hybrid_99 (Table 3). In addition to this, a particular base stacking conformation was found in both models, in which the second thymine was stacking with the first thymine (named HYBRID_P, Figure 4). This conformation was not found in the relevant work of hTel parallel topology [22]. We noted that this TT stacking conformation was not stable and destroyed several times (Figure 5a and Figure 10a). The adenine, which had no special interaction with other bases, did not have a certain conformation in the simulation. The RMSD of this loop was calculated by choosing the experimental structure as the reference (Figure 5b). The value is about 1 Å with tiny fluctuation for hybrid_bsc0, but large fluctuation was found in hybrid_99. It means that the parm99 force field does not have the ability to keep a relatively stable double chain reversal TTA loop.

#### Behavior of Edgewise Loops T
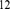
T
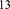
A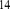 and T
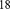
T
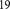
A
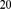


There is a edgewise loop T
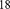
T
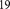
A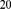 on the upper side of [3+1] hybrid structure, its stacking type was really similar to the lower side (T
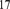
T
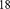
A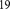 loop) of anti-parallel structure. Firstly, the canonical Watson-Crick base pair T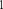/A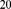 which stacking with the adjacent G-quartet maintained in trajectories (distance and angle between this base pair and G-quartet are listed in Table 2). This TA base pair is stable like the one in anti-parallel structure (the occupied of hydrogen bonds is listed in Table 3). Then, a hydrogen bond between O4 of T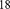 and N2 of G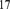 formed and stabilized T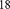, and T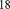 was vertical to the adjacent G-quartet. And then, two conformations of the T
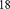
T
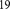
A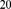 loop were found, in one, T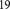 stacked with T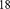 (named HYBRID_U1, Figure 4), and in another, T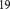 stacked with A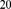 (named HYBRID_U2, Figure 4). In hybrid_bsc0, the T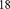/T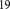 stacking structure existed in 98% of the time, and no long time T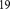/A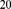 stacking conformation found in the trajectory (Figure 5c). But in hybrid_99, the T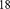/T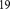 stacking structure was found in just 25% of the snapshots. Both conformations last dozens of nanoseconds, and conformational switch happened several times in hybrid_99 (Figure S3b). MM/PBSA results indicated that HYBRID_U1 is more stable with free energy 36 kcal/mol lower in hybrid_bsc0 (Table 4). It means that T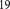 inclines to stack with T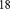 but not A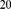, which is different from the anti-parallel T
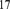
T
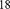
A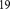 loop.

There is a edgewise loop T
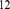
T
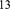
A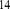 on the lower side of [3+1] hybrid structure. In this loop, the reversed Watson-Crick base pair T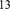/A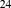 stably stacked with adjacent G-quartet (Table 2 and Table 3), and A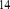 stacked to the adjacent T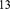/A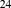 base pair (the conformation named HYBRID_L, Figure 4). The distance and angle and their fluctuations between A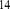 and T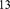/A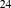 base pair are much bigger than the bases which stacked to the adjacent G-quartet. It means that, this stacking conformation is looser. The last loop base, T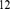 was very flexible, whose behavior likes the T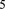 in anti-parallel structure.

#### Behavior of G-stems

Generally, G-stems are rigid part of G-quadruplexes, and G-quartets which four guanine bases dwell in is a plane [30], [31]. But the G-quartet in G-quadruplexes is usually distorted due to the influence of loops and also the dynamical fluctuation of themselves. So, we draw more attention on the distortion and fluctuation of G-quartets here. The parameter RMSD in the Z-axis (RMSD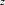) was used to measure the G-quartet distort away from the plane conformation. RMSD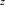 is given by.
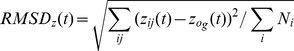
(1)


where 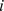 is the base number, 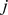 is the atom number, z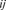 is the z component of the coordinate of atom 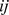, and N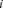 is the number of atoms for base 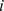.

**Figure 6:**
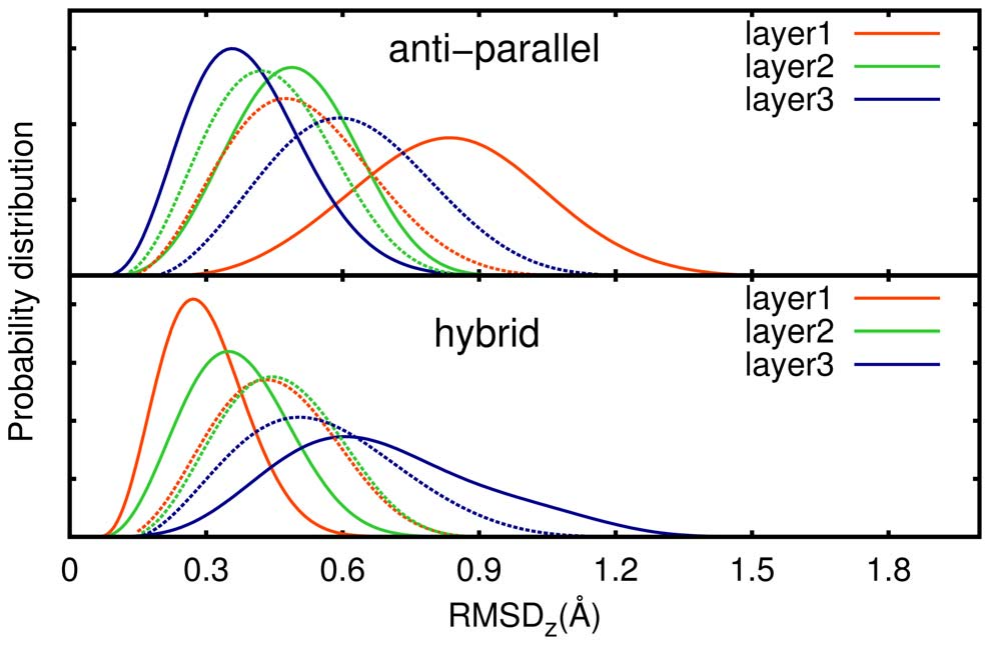
The probability distribution of the RMSD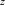 of G-quartets in anti_bsc0 and hybrid_bsc0 models (solid lines), and in anti_stem and hybrid_stem (dash lines).

The probability distribution of RMSD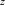 for G-quartets in anti_bsc0 and hybrid_bsc0 are given in Figure 6. As the RMSD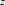 results under parm99 force field are exactly similar to ones in parmbsc0 force field, they are not shown here. What we care about mostly are the peak point and the width of the distribution. Because the peak point represents the distortion of G-quartets, and the width represents the fluctuation of G-quartets. In this part, we care about whether the flexibility of loops contributes to the distortion of G-quartets. In order to prove this, we ran another two simulations in which loop bases are deleted (named anti_stem and hybrid_stem, Table 1). These two simulations just last 5 ns as it's enough for G-stems. We drew the probability distribution of RMSD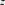 for G-quartets of these two models with dash line in Figure 6. In this figure, we found that the relative distortions and fluctuations are layer1 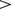 layer2 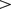 layer3 for anti_bsc0, and layer1 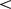 layer2 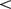 layer3 for hybrid_bsc0. By comparing it with the results of anti_stem and hybrid_stem, we can give some explanation. In anti-parallel structure, the flexible diagonal T
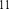
T
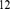
A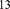 loop led to the deviation of layer1 bigger than the one in anti_stem. On the other side, the stable T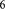/A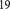 base pair help stabilizing layer3, that it has smaller distortion than the one in anti_stem. In hybrid structure, the stable T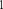/A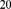 base pair help stabilizing layer1, as it has smaller distortion than in hybrid_stem. On the other side, bigger distortion of layer3 in hybrid_bsc0 comes from the flexibility of edgewise loop T
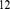
T
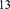
A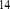, even though there is a T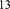/A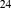 base pair.

One thing make us amazing is that the center layer is not the most stable one, whether in anti_bsc0 or in hybrid_bsc0. It's contrary to the general understanding that central G-quartets which are away from the influence of loops and solvent are more stable than ones in the terminal. In order to explain it, we checked the G-quartets in anti_stem and hybrid_stem. In these two structures, the central one is the most stable one as it is away from the influence of the solvent. So, a brief explanation is that this abnormal phenomenon is the result of the dynamical behavior of loops. In detail, there are two reasons, one is layer3 of anti-parallel structure and layer1 of hybrid structure become more stable by the help of TA base pair, another is layer1 of anti-parallel structure and layer3 of hybrid structure have bigger distortion which comes from the flexibility of adjacent loops, and the central layer is influenced by them. Among this, we can get a conclusion that loops can make the adjacent G-quartet more stable or less stable, base on the behavior of adjacent loops.

## Discussion

Human telomeric G-quadruplex was widely researched by MD simulations, but few people paid attention to the dynamical behavior of loop conformations as it is complicated and changeful. In this work, by the help of the improving of the force field by other groups [20], and also by the help of the new method created to monitor the base stacking conformation in this work, after extended the trajectories to 1 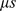, we now have a new insight into the stability and dynamical behaviors of hTel G-quadruplexes TTA loops.

There are distinct stable part and flexible part for edgewise loops and diagonal loops. The stable part, mainly the TA Watson-Crick base pair and most of the bases stacking with G-quartets, is stable enough to maintain in the simulation. Other parts, especially TT base stacking conformations and TA base stacking conformations, are not stable enough to maintain in the simulation, and spontaneous conformational switches happened in our 1 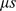 trajectories. It indicates that there are multiple loop conformations with similar energies for these structures. In order to prove this idea, we checked the NMR structure of anti-parallel structure and hybrid structure, and found that in some snapshots of anti-parallel structure, T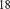 stacked with A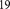, but in other snapshots T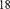 flipped into the bulk solvent. Even though no T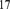/T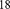 stacking conformation found in these snapshots, it is very likely to form by the help of hydrophobic interaction and 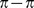 stacking interaction of these two thymine bases. Furthermore, similar free energies also indicate that these two stacking conformations maybe coexist in the solution. There are more details in MM/PBSA results (Table 4), first is that the electrostatic energy (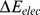) and the polar solvation energy (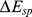) have big deviations with opposite direction. Even though the switch of middle thymine (T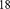 in anti-parallel structure and T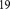 in hybrid structure) also accompanies with the torsion of backbone, which shows in the variation of internal energy (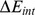), this change is relatively small than 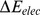 and 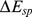. It means that even though the conformation of loops is the result of the balance of these competing forces, but the base stacking conformation is mainly determined by the competition of electrostatic interaction and polar solvation interaction.

The TA Watson-Crick base pair is a unique feature of TTA edgewise loops. All three TA base pairs involved in this work (T
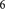
A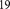 in anti-parallel structure and T
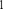
A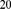, T
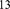
A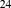 in [3+1] hybrid structure) are very stable. These stable base pairs make a great contribution to steady loop conformations. By the help of these hydrogen bonds interactions, the TTA edgewise loops maintain more stable and simple conformations than T3 and T4. As the TTA edgewise loops mainly maintained their experimental conformation and only single thymine base was flapping in the hundreds of nanoseconds simulation, while the experimental conformation of T3 and T4 were almost always lost and diverse conformations were found in previous simulations [18], [22]. And also, it's understandable that the TTA loops have more stable conformation than AAA loops, and TTA replaced with AAA led to the melting temperature of the G-quadruplex decreased [5].

Double chain reversal loops did not maintain a single stable conformation in the simulation. It is similar to the relevant work of the parallel structure of hTel [22]. In our opinion, there are two reasons for this unstability. On one hand, loop conformation is the balance of multiple interactions, and this balance is extremely hard to reach under the ability of present force fields for double chain reversal loops [22]. On the other hand, indeed the double chain reversal loop has multiple conformations with similar free energies. So, this loop will not maintain a certain conformation in the simulation. This view can be proofed by that the initial structure of the double chain reversal loop, which is given by the NMR experiment, also don't has a certain conformation [26]. All these results reflect the unstability of double chain reversal loops.

Besides, we should note that the stability of loop conformations discussed above does not directly relate to the relative stabilities of these two G-quadruplexes topologies. Actually, due to the different sequences and sequence lengths of hybrid structure (d[T
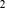
G
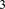
(T
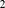
AG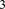)
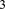
A]) and anti-parallel structure (d[AG
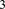
(T
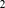
AG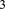)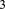]), we think it is not possible to compare the relative stabilities of these two structures base on the results of MM-PBSA and MD simulations directly.

## Methods

### Molecular Dynamics Simulations

The AMBER force field parmbsc0 was used in this work as it works well for simulating nucleic acids [29]. Besides, the parm99 force field was chosen to make a comparison. As we realized the limitation of these force fields [22], we did simulations carefully and the results were compared to the experimental structures to make sure that they are valid.

The models were built by *tleap* program in AMBERTOOLS 1.5 software package [32]. The *acpype* program [33] was used to convert the AMBER file formats to GROMACS file formats, and single precision GROMACS 4.5 software package [34] was used to run the simulations.

Explicit solvent simulations were performed at 300 K under the control of Berendsen thermostat with time constant 1 ps [35]. Isotropic constant-pressure boundary condition under the control of the Berendsen algorithm of pressure coupling with time constant 1 ps was used for NPT ensemble simulations. The Particle mesh Ewald (PME) method was used for calculating electrostatic interactions [36]. A 10 Å cut-off was applied to Lennard-Jones interactions. A cubic box of TIP3P water molecules were added around the DNA to a depth of 12 Å on each side of the solute [37]. The system was neutralized with the addition of potassium ions or sodium ions, and two of them were placed between the three G-quartets layers. Standard AMBER parm94 ion parameters were used here. Covalent bonds contain hydrogen atoms were constrained using the SHEAK algorithm [38]. The time step for integration in all simulations was 2 fs. Coordinates were written to trajectory files every 5 ps. Periodic boundary conditions were applied to avoid surface effects.

An energy minimization of 1000 steps using the steepest descent algorithm was followed by a 20 ps position-constrained MD simulation in order to equilibrate water and ions, and then, 200 ps additional simulation was add under NPT ensemble. At last, production simulations were run under NVT ensemble.

### Free Energy Calculation

The Molecular Mechanic/Poisson-Boltzmann Surface Area (MM/PBSA) method was chosen to calculate the free energy [39]. The solvation free energy was computed as sums of polar and nonpolar contributions using APBS software [40]. The MM energy parts were calculated in GROMACS. The solute entropic part was calculated by normal mode analysis, and *mdrun* and *g
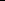
nmeig* in GROMACS were used to calculate eigenvalues. All water molecules and cations (except the two locate in the center of the G-quadruplex) were removed from the trajectories. Standard AMBER parm94 ions radius were used here. The trajectories were examined in 50 ps intervals. Values used for the dielectric constant were 1 for solute and 80 for the surrounding solvent. The radius of the solvent was set to 1.4 Å.

### Conformational Analysis

**Figure 7:**
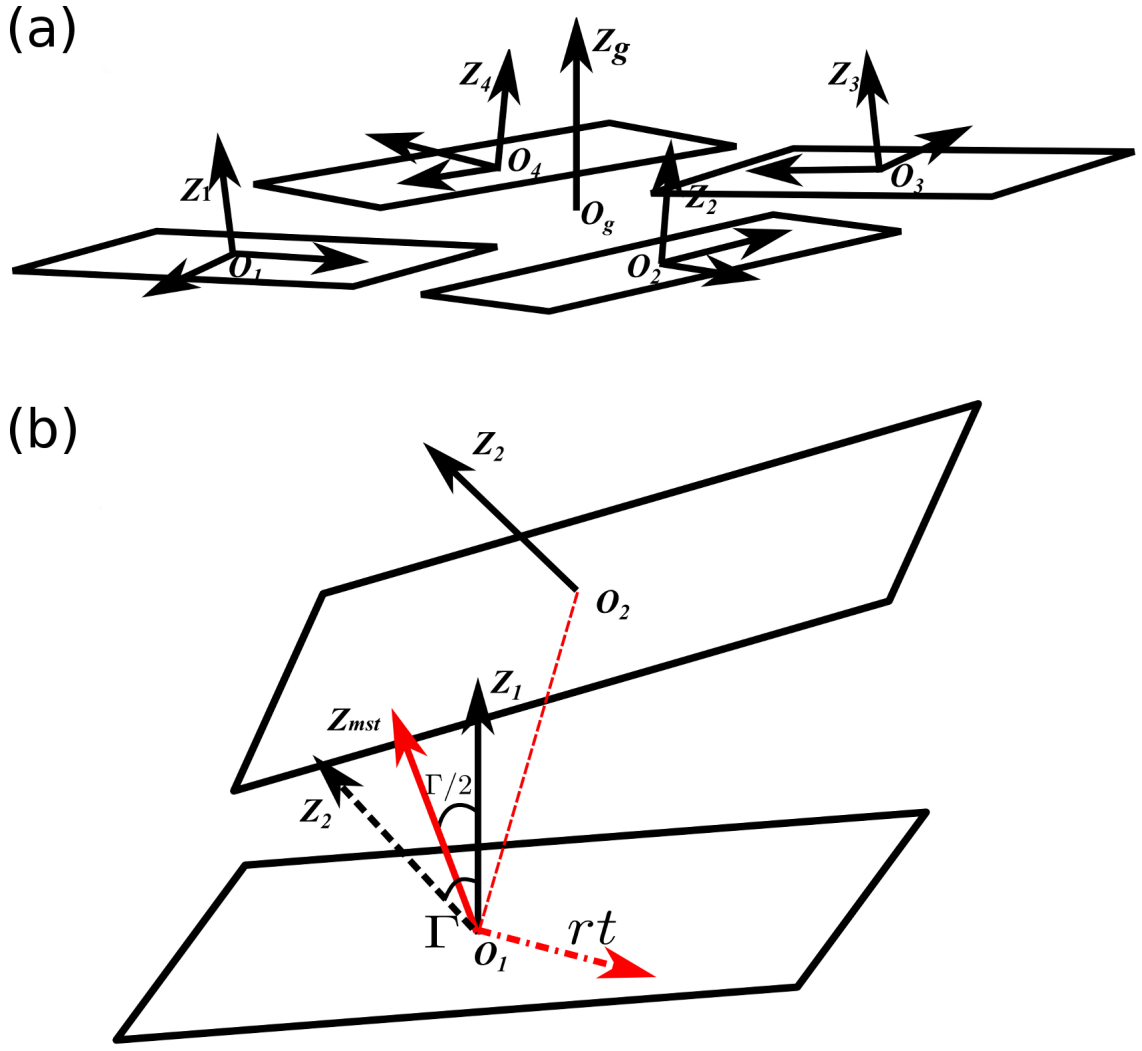
Illustration of the G-quartet base-triads. (a), G-quartet base-triads. All four bases have similar Z-axis but distinct X-axis and Y-axis. (b), Calculating for Z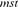 by rotating the Z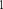 by 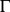/2 about the *rt* axis, and 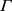 is the angle between two Z-axes Z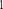 and Z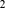.

A G-quartet consisting of four guanines shown in Figure 7a usually does not stay in the same geometrical plane during the dynamic processes due to deformation of the G-quadruplex. In order to calculate the distance and angle between G-quartet and loop base, a center point O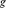 and a Z-axis Z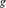 are used to represent the G-quartet in our model, and the algorithms are established based on the strategy applied to the description of the double stranded DNA base and base pairs. Details are given below.

The coordinates of the base are described by the base-triad base on El Hassan's work [41]. Then by using a least squares fitting procedure, the real base is fit to a standard reference base, the center of bases, O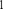, O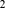, O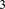 and O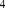, and Z-axes, Z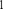, Z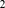, Z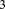 and Z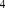 which perpendicular to bases (for a standard base, all the non-hydrogen atoms are in the XY plane) can be calculated. Figure 7a is an illustration of the base-triad for G-quartet, it was found that four base-triads have similar Z-axis but different X-axis and Y-axis. In the following, the four Z-axes, Z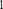, Z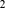, Z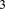 and Z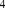, are associated to create a new Z-axis, Z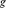.

First of all, we give an example to show the procedure for creating a new Z-axis, Z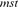, via association of two unparallel Z-axes, say Z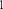 and Z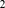. The angle 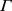 between two axes Z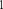 and Z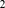 can be calculated using the formula below:
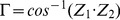
(2)


Two bases with similar Z-axis can be associated to create a 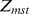 vector by rotate the base 1 by 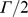 about the 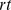 axis:
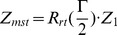
(3)



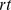
 is the rotate axis determined by cross product two Z-axes:
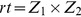
(4)



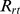
 is the general rotation matrix, and 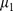, 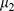, 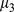 are three components of unit vector 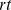:
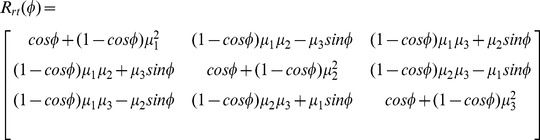
(5)


This algorithm refers to Lu's work [42] and the 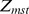 represents the Z-axis of the two bases (Figure 7b).

The Z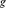 for G-quartet is created in the same principle. The four guanine bases in quartet are divided into two parts, each Z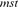 is created by rotating the first base by 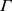/2 about the 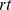 axis in each part. In this way it will produce two values of Z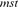. Finally, two generated Z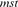 are associated together to create a new Z-axis, Z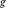, by rotating the base corresponding to one of two Z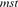.

Centers of four guanine bases are associated to create a new center 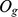 which represents the center of the G-quartet as well.
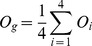
(6)


Now, the angle 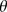 and distance in Z-axis 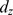 between two G-quartets or between G-quartet and loop base can be calculated directly after we get the Z-axis and center of two base groups (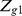, 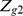, 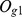, 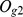).
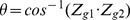
(7)



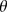
 is the angle between two plane base groups.

The two Z-axes are combined to create a 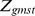 axis:
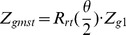
(8)


Then we can get the distance of 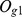, 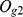 in Z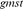 axis d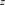, it's the distance in Z-axis for two plane base groups.
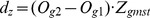
(9)


In this work, we used the distance and angle (and also their fluctuation) between two bases, or between base and adjacent G-quartet to monitor the stability of stacking structures. We assumed that if the distance maintains in the range of 3 Å to 4 Å, and the angle maintains in the range of 0 to 30 degrees, then it forms a stacking structure.

MDAnalysis software [43] and some *in-house* programs were been used for data analysis. Standard reference data of nucleic bases came from Olson's work [44]. The base fitting algorithms in 3DNA [45] have been used in our methods. *ptraj* in AmberTools 1.5 was used to calculate the lifetime and occupied of hydrogen bonds, and the cut-off of distance and angle are 3.5 Å and 30 degrees.

## Supporting Information

Figure S1 **RMSD of G-stems for all models.**
(TIF)

Figure S2
**Structural dynamics of anti_99 loops.** (a), distance and angle between two thymine bases T_11_ and T_12_ of the upper diagonal loop, the big fluctuation reflects that no stable stacking conformation formed. (b), distance and angle between A_7_ and upper G-quartet. A_7_ stacked with G-quartet in two periods, which are from 0 ns to 100 ns and from 620 ns to 750 ns. (c), angle between T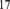 and T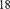 and angle between T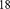 and A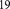.(TIF)

Figure S3
**Structural dynamics of hybrid_99 loops.** (a), distance and angle between two thymine bases T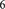 and T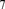 of double chain reversal loop. (b), the angle between T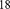 and T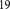 and angle between T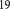 and A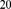.(TIF)

Table S1 **Distance and angle between loop bases and their reference base groups.**
(DOC)

Table S2 **Details of hydrogen bonds between loop bases and between loop base and quartet base.**
(DOC)

Table S3 **Comparison of the absolute free energies of the different conformations of the loops.**
(DOC)
