# Supplementary figures and images for: Structural Dynamics of Human Telomeric G-Quadruplex Loops Studied by Molecular Dynamics Simulations
Source: PLoS One. 2013 Aug 8;8(8):e71380. doi: 10.1371/journal.pone.0071380 (PMC3738534; doi:10.1371/journal.pone.0071380)

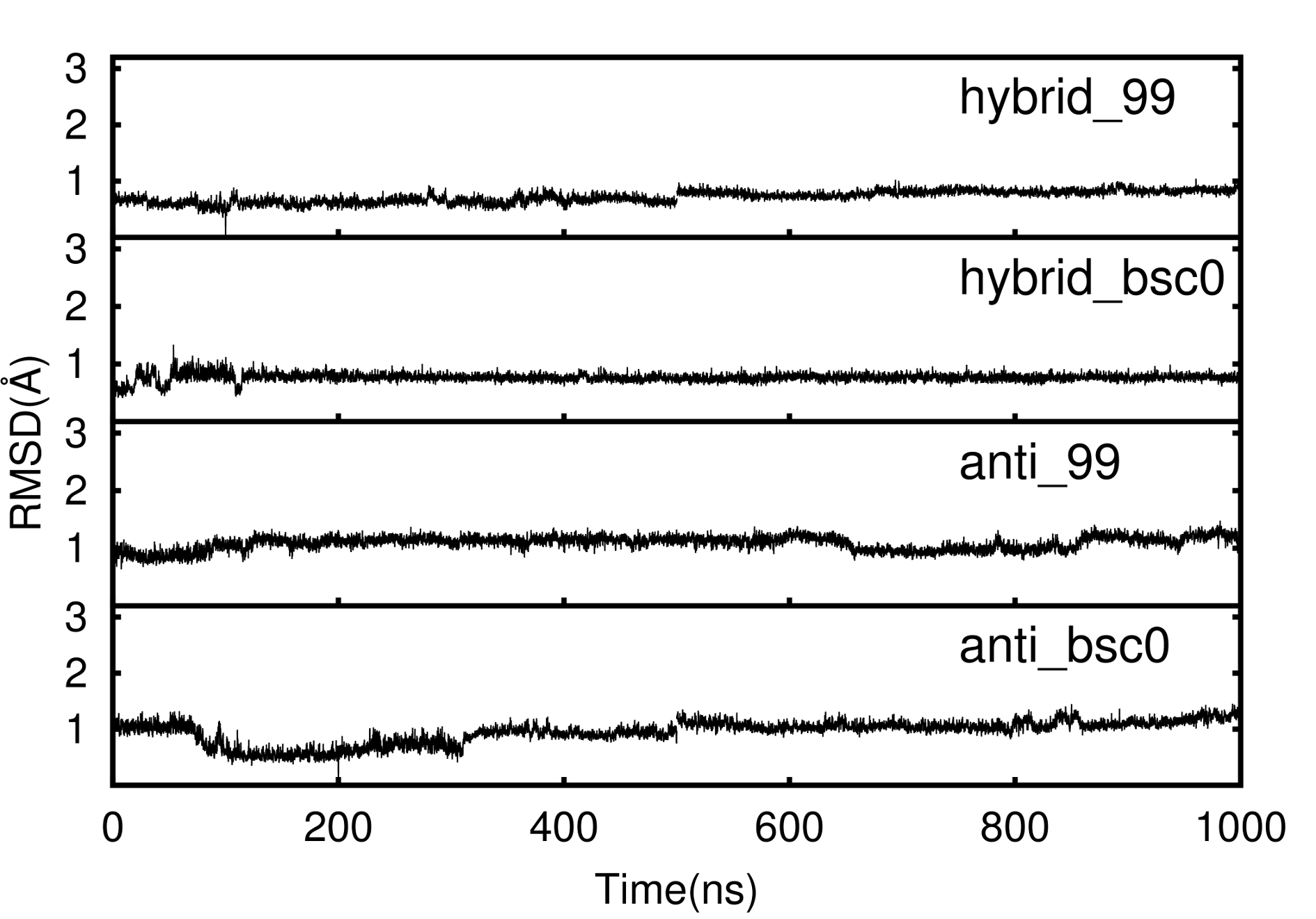

Supplement: Figure S1 — RMSD of G-stems for all models. (TIF) [file pone.0071380.s001.tif]

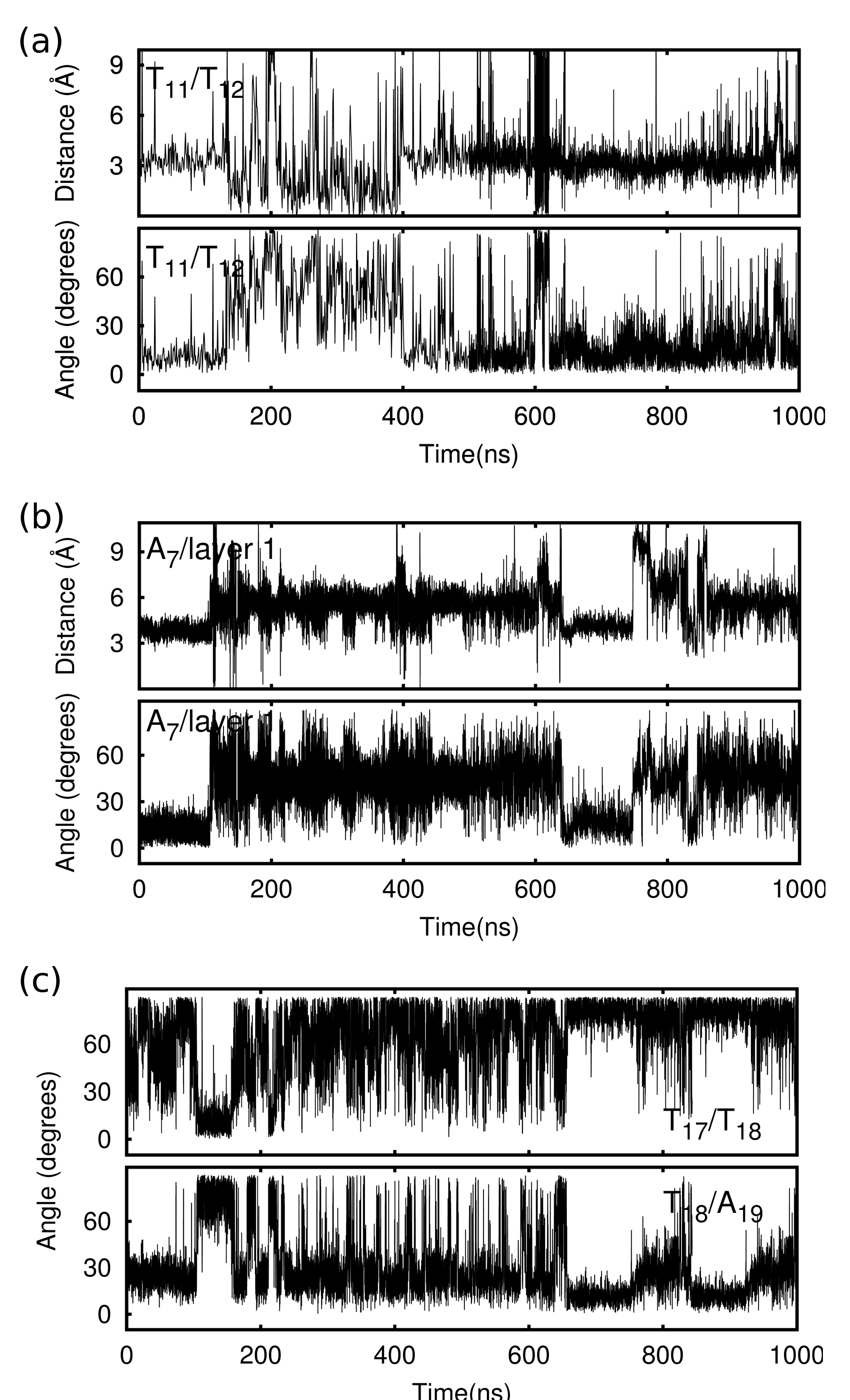

Supplement: Figure S2 — Structural dynamics of anti_99 loops. (a), distance and angle between two thymine bases T11 and T12 of the upper diagonal loop, the big fluctuation reflects that no stable stacking conformation formed. (b), distance and angle between A7 and upper G-quartet. A7 stacked with G-quartet in two periods, which are from 0 ns to 100 ns and from 620 ns to 750 ns. (c), angle between T and T and angle between T and A. (TIF) [file pone.0071380.s002.tif]

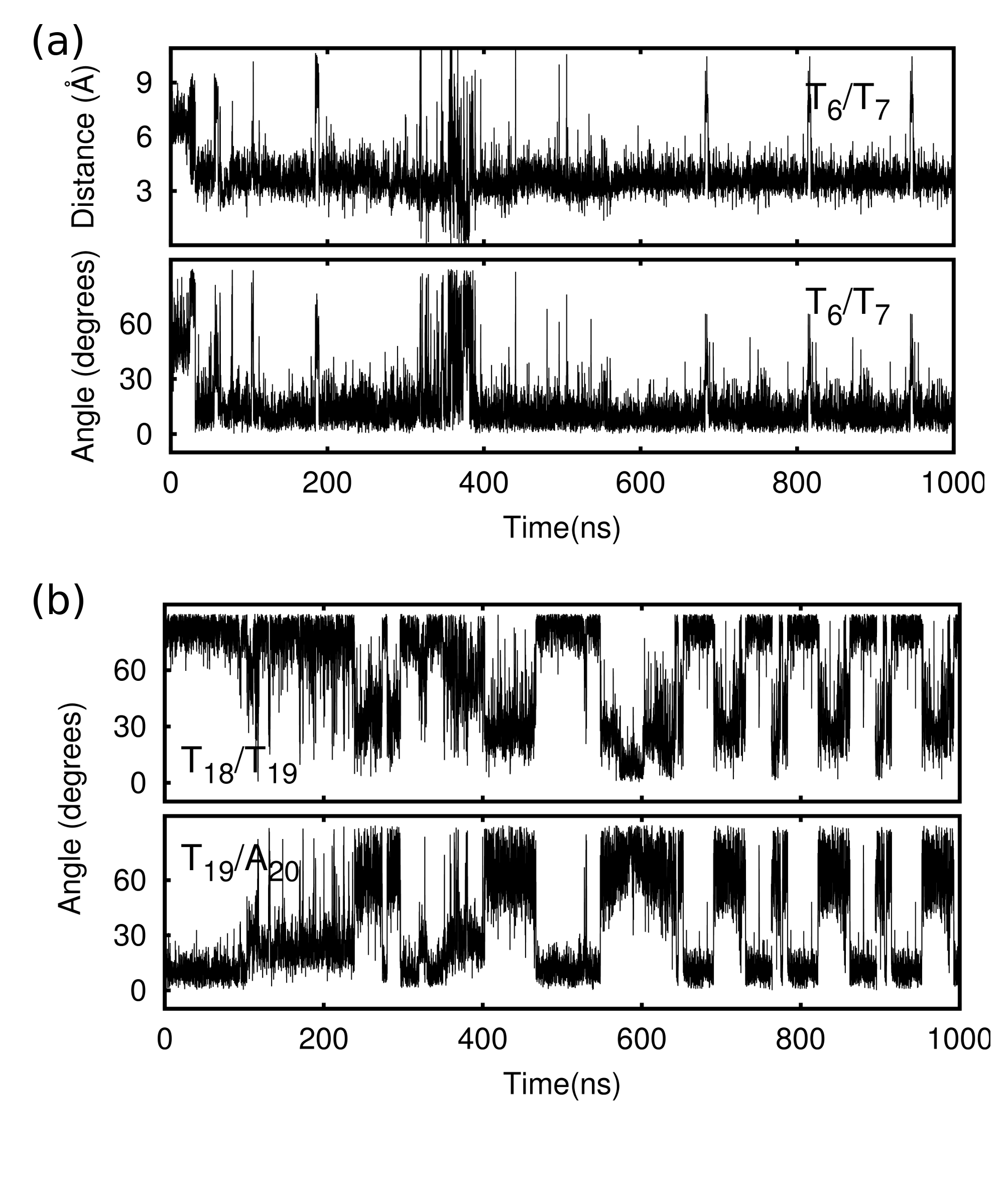

Supplement: Figure S3 — Structural dynamics of hybrid_99 loops. (a), distance and angle between two thymine bases T and T of double chain reversal loop. (b), the angle between T and T and angle between T and A. (TIF) [file pone.0071380.s003.tif]
