# Supplementary material for: Structural Dynamics of Human Telomeric G-Quadruplex Loops Studied by Molecular Dynamics Simulations
Source: PLoS One. 2013 Aug 8;8(8):e71380. doi: 10.1371/journal.pone.0071380 (PMC3738534; doi:10.1371/journal.pone.0071380)
Supplement: Table S1 — Distance and angle between loop bases and their reference base groups. (DOC) [file pone.0071380.s004.doc]

Table S1: Distance and angle between loop bases and their reference base groups^a^.

| Model | Group 1 | Group 2 | Distance(Å) | Angle(degree) |
| --- | --- | --- | --- | --- |
| anti_99 | A_1_ | Layer 1 | 3.0±0.4 | 12.2±6.6 |
|  | A_13_ | Layer 1 | 3.9±0.8 | 17.7±17.9 |
|  | T_6_ | Layer 3 | 2.8±0.5 | 12.5±6.0 |
|  | A_19_ | Layer 3 | 2.9±0.4 | 14.6±6.6 |
| hybrid_99 | T_1_ | Layer 1 | 2.6±0.4 | 13.2±5.8 |
|  | A_20_ | Layer 1 | 2.9±0.3 | 11.0±5.9 |
|  | T_13_ | Layer 3 | 2.6±0.4 | 16.1±8.2 |
|  | A_24_ | Layer 3 | 2.7±0.4 | 17.6±9.2 |
|  | A_14_ | T13/A24 | 4.8±0.8 | 26.8±11.1 |

^a^The adjacent G-quartet or adjacent base pair are chosen as the reference base groups.
