# Supplementary material for: Structural Dynamics of Human Telomeric G-Quadruplex Loops Studied by Molecular Dynamics Simulations
Source: PLoS One. 2013 Aug 8;8(8):e71380. doi: 10.1371/journal.pone.0071380 (PMC3738534; doi:10.1371/journal.pone.0071380)
Supplement: Table S2 — Details of hydrogen bonds between loop bases and between loop base and quartet base. (DOC) [file pone.0071380.s005.doc]

Table S2: Details of hydrogen bonds between loop bases and between loop base and quartet base.

| Model | Donors | Acceptors | Occupied(%) | lifetime(ps) |
| --- | --- | --- | --- | --- |
| anti_99 | N6@A_19_ | O4@T_6_ | 98.4 | 353.0 |
|  | N3@T_6_ | N1@A_19_ | 94.3 | 138.5 |
|  | N2@G_8_ | O4’@A_7_ | 94.4^a^ | 103.5 |
| hybrid_99 | N3@T_13_ | N1@A_24_ | 97.6 | 548.5 |
|  | N6@A_24_ | O2@T_13_ | 99.7 | 2056.5 |
|  | N3@T_1_ | N1@A_20_ | 99.7 | 11762.0 |
|  | N6@A_20_ | O4@T_1_ | 96.4 | 193.5 |
|  | N2@G_4_ | O4@T_6_ | 43.1 | 50.0 |

^a^This H-bond formed after 100 ns in the simulation, in order to make it consist with Table 3, just last 700 ns of the trajectory was been used to count the H-bond.
