# Supplementary material for: Structural Dynamics of Human Telomeric G-Quadruplex Loops Studied by Molecular Dynamics Simulations
Source: PLoS One. 2013 Aug 8;8(8):e71380. doi: 10.1371/journal.pone.0071380 (PMC3738534; doi:10.1371/journal.pone.0071380)
Supplement: Table S3 — Comparison of the absolute free energies of the different conformations of the loops. (DOC) [file pone.0071380.s006.doc]

Table S3: Comparison of the absolute free energies^a^ of different conformations of loops.

| Topologies | Conformations | ΔE_int_ | ΔE_elec_ | ΔE_vdw_ | ΔE_sp_ | ΔE_nsp_ | ΔPB | ΔTS | ΔG |
| --- | --- | --- | --- | --- | --- | --- | --- | --- | --- |
| anti_99 | ANTI_L1-ANTI_L2 | -3 | 85 | -4 | -91 | 0 | -14 | -1 | -13 |
| anti_99 | ANTI_L4-ANTI_L3 | -10 | -14 | 2 | 16 | 0 | -6 | 1 | -7 |
| hybrid_99 | HYBRID_L1-HYBRID_L2 | 26 | -90 | -12 | 57 | 1 | -18 | 4 | -22 |

^a^ΔE_int_, internal energy, ΔE_elec_, Coulombic energy, ΔE_vdw_, van der Waals energy, ΔE_sp_, polar solvation energy, ΔE_nsp_, nonpolar solvation energy, ΔPB, enthalpy, ΔTS, solute entropy, ΔG, absolute free energy. All are in Kcal/mol.
